# Early-life famine exposure increases the risk of subsequent physical disability: evidence from a national population-based survey

**DOI:** 10.3389/fpubh.2025.1587680

**Published:** 2025-07-09

**Authors:** Shiwei Cao, Yao Wu, Tengfei Niu, Xiyu Chen, Jie Xiang, Hao Li, Ziyi Chen, Qianying Shi, Yu Du

**Affiliations:** ^1^The Second Clinical College, Chongqing Medical University, Chongqing, China; ^2^International Medical College, Chongqing Medical University, Chongqing, China; ^3^Department of Basic Courses, Chongqing Medical and Pharmaceutical College, Chongqing, China; ^4^The First Clinical College, Chongqing Medical University, Chongqing, China; ^5^College of Foreign Languages, Chongqing Medical University, Chongqing, China; ^6^College of Traditional Chinese Medicine, Chongqing Medical University, Chongqing, China; ^7^The Fifth Clinical College, Chongqing Medical University, Chongqing, China; ^8^Department of Orthopedic Surgery, The Second Affiliated Hospital of Chongqing Medical University, Chongqing, China

**Keywords:** early-life famine exposure, physical disability, malnutrition, CHARLS, middleaged and older adults

## Abstract

**Background:**

Due to ethical constraints, famines, which can lead to severe malnutrition, are often viewed as natural experiments to assess the impact of early-life nutritional malnutrition on adverse health outcomes in adulthood. However, evidence regarding the effect of early-life famine exposure on later-life physical disability remains scarce. This study aims to investigate the association between early-life famine exposure and physical disability in the Chinese population.

**Methods:**

This study is based on survey data from the China Health and Retirement Longitudinal Study released in 2018. Famine exposure was determined based on participants’ birth years, and all individuals were categorized into four groups: non-exposed, fetal exposed, preschool exposed, and school-age exposed. Logistic regression analysis was conducted to examine the association between early-life famine exposure and physical disability. Stratified analyses were further performed by gender, residence, and severity of early-life famine exposure.

**Results:**

After adjusting for all covariates, fetal exposure to famine was associated with a 1.57-fold increased risk of severe disability compared to the non-exposed group. Among males, older people with fetal exposure to famine was significantly associated with a 1.69-fold increased risk of severe disability compared to the non-exposed group. Among females, older populations with famine exposure during the school-age period was associated with a 1.52-fold increased risk of severe disability compared to the non-exposed group. In areas with less severe famine exposure, those with fetal exposure was linked to a 1.66-fold increased risk of severe disability compared to the non-exposed group. In areas with more severe famine exposure, older individuals exposed during the preschool period had a 1.57-fold higher risk of mild disability. In rural areas, older adults who were exposed to famine during the fetal period had a 1.65-fold increased risk of severe disability compared to their non-exposed counterparts. In urban areas, those exposed to famine during the preschool period exhibited a 1.63-fold higher risk of mild disability than their non-exposed counterparts.

**Conclusion:**

The findings underscore the critical role of early-life adversity and nutritional status in shaping health outcomes in later life, highlighting the need for public health policies to prioritize nutritional interventions during early developmental stages.

## 1 Introduction

According to the developmental origins of health and disease (DOHaD) hypothesis, adversity in early life may affect growth, metabolism and neurodevelopment, thus leading to early adaptations of body structure and function, which may facilitate short-term survival but raise the risk of adverse health outcomes in the long run (1). Adversity experienced in early life not only exerts a direct impact on physical health but also spills over into social factors such as mental health and socioeconomic status, which in turn influences an individual’s access to healthcare resources as well as what health challenges they will face in the future (2). Over time, the cumulative effect of these health resources or risks may exacerbate health inequalities among individuals.

For ethical reasons, few studies have directly assessed the relationship between early-life adversity and adverse health outcomes. Many researchers have used external shocks such as wars, famines, natural disasters, and economic recessions as proxies for early-life adversity to investigate their effects on adverse health outcomes (3–5). A recent article published in *The Lancet* explored the impacts of wildfires and heatwaves on early childhood physical health, mental health, and neurodevelopment (5). A 2021 review highlighted that natural disasters such as floods, earthquakes, tsunamis, and hurricanes increase the burden of long-term metabolic health damage (6). Another review exploring the relationship between famine exposure and health outcomes found that early famine exposure (EFE) was associated with physical size, diabetes, and schizophrenia (7). The long-term effects of early malnutrition on fetal, infant, and child physical and mental health have been extensively reported (8–10). A study by Ting Wu et al. showed that EFE is associated with a higher risk of possible sarcopenia in older adults (11). Moreover, a recent meta-analysis also revealed that EFE is linked to osteoporosis and low bone mineral density, particularly among females and older adults (12). Previous studies have confirmed a significant association between sarcopenia, osteoporosis, and physical disability (13, 14). It is evident that EFE may contribute to an increased risk of physical disability to some extent.

Physical disability in middle-aged and older adults seems inevitable as they get older (15). Globally, about 1 billion people are reported to suffer from disabilities, and this number is still increasing as aging exacerbates (16). In China, approximately one in five older adults have a disability (17). A substantial body of research findings indicates that disability is associated with low quality of life, obesity, depression, chronic diseases, and an increased risk of death (18–21). Additionally, disability imposes considerable economic costs on families and healthcare systems (22). Studying the impact of EFE on disability deeply is therefore critical to delineating priority intervention populations and developing clinical care strategies to reduce the adverse effects of disability.

The Great Chinese Famine (1959–1961) provides a natural experimental context to study the effects of early-life malnutrition on adverse health outcomes in adulthood (23). The Great Famine, caused by a combination of natural disasters and policy errors during the Great Leap Forward (GLF), is also known as the GLF Famine (24). It is noteworthy that the Great Chinese Famine was a nationwide catastrophe rather than a regional event, and it is widely considered the most severe famine in recorded human history (25). During this period, nearly all of mainland China suffered from extreme food deprivation, leading to an estimated 30 million deaths and a mortality rate of over 3.0% (26).

Notably, the effects of EFE on adverse health outcomes may vary by gender. For instance, recent literature suggests that prenatal malnutrition leads to physical dysfunction in late adulthood, particularly in males, but not in females (27). Moreover, different residences may be characterized by varying socioeconomic backgrounds, healthcare resources, and living conditions (28), which could act as moderating factors in the long-term health outcomes following early-life famine exposure. Previous studies have also shown that in regions severely affected by famine, EFE significantly increases the risk of adult-onset diabetes and metabolic syndrome, whereas this effect does not occur in areas with less severe famine exposure (29, 30). This suggests that the severity of famine exposure may lead to different health outcomes. Therefore, it is important to explore the association between EFE and physical disability under different genders, residence and famine severity conditions.

In summary, this study aims to analyze the association between EFE and physical disability using cross-sectional survey data from the China Health and Retirement Longitudinal Study (CHARLS) released in 2018. And this study also further explores the specific associations under stratifications by gender, residence and famine severity.

## 2 Materials and methods

### 2.1 Population

The data used in this study were derived from the cross-sectional dataset of the CHARLS released in 2018. CHARLS is a nationally representative survey designed to collect comprehensive information on the health and aging of adults aged 45 years and older in China. CHARLS covers 28 provinces, 150 counties, and 450 villages. Since its initiation in 2011, the survey has been conducted every 2–3 years (31). CHARLS was approved by the Ethics Review Committee of Peking University, and all participants provided written informed consent (32). The study followed the Strengthening the Reporting of Observational Studies in Epidemiology (STROBE) guidelines for reporting (33).

**Figure 1:**
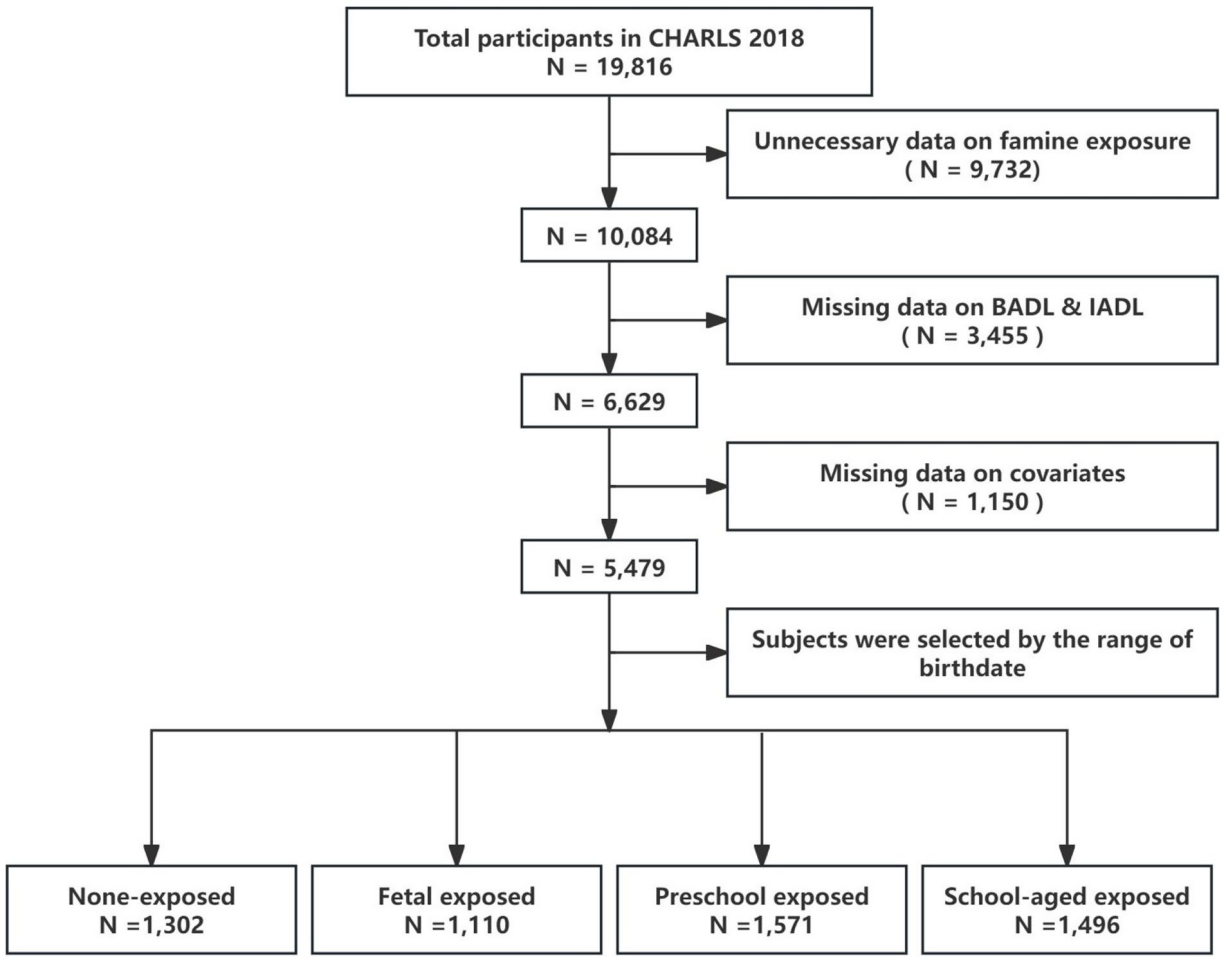
Flowchart for the study design and participants.

A total of 19,816 individuals participated in the 2018 follow-up survey. We included 10,084 participants born within the following periods: (1) January 1, 1950 to December 31, 1957; (2) January 1, 1959 to December 31, 1962; (3) January 1, 1964 to December 31, 1967. We excluded 3,455 participants who lacked data on Basic Activities of Daily Living (BADL) and Instrumental Activities of Daily Living (IADL), as well as 1,028 participants with missing covariate information. Ultimately, 5,479 participants were included in the analysis, with the detailed selection process shown in Figure 1.

The sample size for this study was determined using the formula for calculating sample size in cross-sectional studies $n=(Z_{\alpha /2}^{2}*p*q)/\delta^{2}\;$ (34): (1) n represents the required sample size; (2) p denotes the prevalence rate of IADL or BADL among older Chinese adults; (3) q = (1 - *p*); (4) Z_*α*/2_ was set at 1.96, and *α* was set at 0.05 for the two-sided test; (5) *δ* denotes the allowed error, which was calculated as 0.1*p*. According to a previous study, the prevalence of BADL and IADL among older Chinese adults was 34.3 and 50.5% (35), which indicated that at least 736 and 377 participants were needed, respectively, to meet the required sample size. We included 5,479 participants, which is substantially greater than the minimum required sample size, thus ensuring that the sample size for this study is adequate.

### 2.2 Grouping

Given that famine status peaked between 1959 and 1962 (23), we adopted the birth year to measure famine exposure across different periods and divided all participants into four groups: non-exposed (born January 1, 1964 to December 31, 1967), fetal exposed (born from January 1, 1959 to December 31, 1962), preschool exposed (born from January 1, 1954 to December 31, 1957), and school-age exposed (born from 1950 January 1 to December 31, 1953) (36).

### 2.3 Assessment of physical function

The Basic Activities of Daily Living Scale and Instrumental Activities of Daily Living Scale were utilized to assess one’s physical functioning (37, 38). BADL s are skills that meet basic physical needs, including dressing, bathing, eating, getting up and off the bed, going to the toilet, and controlling urination and defecation. IADLs are activities that reflect the ability to live independently, including housework, food preparation, shopping, financial management, and taking drugs. ADL restriction is defined as the inability to independently perform any one or more of the BADL or IADL, and are named BADL restriction and IADL restriction, respectively (39). Disability status was grouped into three levels: patients who could complete all BADL and IADL independently had no disability; those who could complete BADL items independently but needed assistance in completing IADL items had mild disability; and those who could not complete BADL tasks independently had severe disability (40). In the present study, the Cronbach’s alpha for the IADL and BADL scales were 0.833 and 0.831, respectively.

### 2.4 Assessment of covariates

Sociodemographic, behavioral characteristics, and health-related data from CHARLS were used as covariates in this study to control for potential confounding biases. These covariates include age, gender, education level, residence, living arrangement, self-rated economic situation, smoking, drinking, exercise, self-rated health, life satisfaction, cognitive impairment, depressive symptom, hypertension, diabetes, stroke, osteoarthritis, altitude, and famine severity.

Education level was split into three groups: illiteracy, primary school or below, and secondary school or below. Residence was categorized as urban and rural, and living arrangement was classified as living with family and others. Economic situation was self-rated as wealthy, fair, or poor. Smoking and drinking were categorized as yes and no. Exercise was divided into four categories based on the level of physical activities per week: no exercise, low intensity (e.g., walking, etc.), average intensity (e.g., lifting light objects, bicycling at a regular speed, etc.), and high intensity (e.g., lifting heavy objects, digging, and plowing, etc.). Self-rated health was categorized as good and bad, and life satisfaction was classified as satisfaction and dissatisfaction. Depressive symptoms and cognitive impairment were dichotomized according to the CESD-10 and MMSE scales, respectively. Chronic diseases (hypertension, diabetes, stroke and osteoarthritis) were categorized as yes and no. Previous studies have shown a higher prevalence of disability among middle-aged and older adults living at high altitude, possibly due to chronic hypoxia and malnutrition, which exacerbate age-related declines in physiological reserve, leading to greater susceptibility to disability as a result of impaired muscle function (41, 42). So we divided China into three parts according to regional average altitude: regions with an average altitude of more than 4,000 m above sea level, regions with an average altitude of 1,000–2,000 m above sea level, and regions with an altitude of less than 500 m above sea level (43).

In addition, despite the fact that the whole mainland China suffered from famine, the severity of famine varied from region to region due to differences in regional climate, local policies, and population density. Referring to previous studies (44), we defined the severity of famine with the excess mortality rate, namely the percentage change from the highest mortality rate in 1959–1961 to the average mortality rate in 1956–1958. In this study, the excess mortality rate of 50% was employed to distinguish between two levels of famine: provinces with an excess mortality rate of less than 50% were considered as mild famine areas, and provinces with an excess mortality rate of more than 50% were regarded as severe famine areas (44).

### 2.5 Statistical analysis

The Kolmogorov–Smirnov test was used to assess the normality of continuous variables. Continuous variables that followed a normal distribution were presented as mean ± standard deviation (M ± SD), while categorical variables were presented as frequencies and percentages. Analysis of variance (ANOVA) and chi-square (*χ*^2^) tests were used to compare differences between groups. Logistic regression analysis was performed to determine the association between EFE and physical disability. Model 1 is a crude model. Model 2 adjusted for age, gender, residence, marital status, education level, smoking, drinking, exercise, self-rated health, life satisfaction, altitude, famine severity, cognitive impairment, depressive symptom, hypertension, diabetes, and stroke. Odds ratios (ORs) and 95% confidence intervals (CIs) were reported for all regression models. The goodness-of-fit of the logistic regression models was assessed using the Hosmer-Lemeshow test, with a *p*-value greater than 0.05 indicating a good fit. The linearity assumption for continuous variables in the logistic regression model was tested using the Box-Tidwell procedure. First, the natural logarithms of the continuous variables were calculated, and the interaction terms of the original variables and their natural logarithms were added to the model. If the interaction term *p*-value was less than 0.05, it was concluded that there was no linear relationship between the variable and the log-odds of the outcome. Given the differential impact of EFE on males and females, as well as variations in resource allocation by residence and the severity of famine exposure, stratified analyses were conducted by gender, residence, and famine severity. Additionally, two sensitivity analyses were conducted to assess the robustness of the results. First, missing data were handled using chained equations to ensure a comprehensive examination of the dataset. Second, recognizing the unreliability of self-reported data from older adults with cognitive impairment, participants with cognitive impairment were excluded from the analysis. All statistical analyses were performed using SPSS version 27.0. A two-sided *p*-value of <0.05 was considered statistically significant.

## 3 Results

### 3.1 Basic characteristics of the study population

The mean age of the 5,479 participants involved was 60.20 ± 5.38 years old, with 2,769 (50.54%) females and 2,710 (49.46%) males (Table 1). A total of 302 individuals were unexposed to EFE, while 20.25, 28.67, and 27.30% of participants were exposed during the fetal, preschool, and school-age periods, respectively. ANOVA results revealed a significant association between age and EFE (*p* < 0.05), with a progressive increase in mean age observed across the unexposed, fetal, preschool, and school-age exposed groups. Chi-square tests indicated significant associations between EFE and gender, education level, residence, living arrangement, economic situation, exercise, self-rated health, life satisfaction, cognitive impairment, stroke, and altitude (*p* < 0.05). Among individuals exposed to famine during the school-age period, the prevalence of disability was higher in males than in females. In contrast, within the unexposed group and those exposed during the fetal and preschool periods, females exhibited a higher prevalence of disability than males. Rural participants exposed to famine during the preschool and school-age periods had a higher prevalence of disability compared to their urban counterparts. Additionally, in regions less severely affected by the famine, the prevalence of disability was higher among those exposed during the fetal and school-age periods than among those in more severely affected areas.

**Table 1:** Characteristics of participants by period of famine-exposure.

Variables	Total (*n* = 5,479)	Non- exposed (*n* = 1,302)	Fetal exposed (*n* = 1,110)	Preschool exposed (*n* = 1,571)	School-age exposed (*n* = 1,496)	*p*
Age, Mean ± SD	60.20 ± 5.38	52.63 ± 1.08	57.32 ± 1.16	62.55 ± 1.12	66.47 ± 1.10	**<0.001**
Gender, *n* (%)						**<0.001**
Male	2,710 (49.46)	591 (21.81)	494 (18.23)	754 (27.82)	871 (32.14)	
Female	2,769 (50.54)	711 (25.68)	616 (22.25)	817 (29.51)	625 (22.57)	
Education level, *n* (%)						**<0.001**
Illiteracy	3,546 (64.72)	773 (21.80)	556 (15.68)	1,034 (29.16)	1,183 (33.36)	
Primary school or below	1,303 (23.78)	416 (31.93)	328 (25.17)	339 (26.02)	220 (16.88)	
Secondary school or below	471 (8.60)	69 (14.65)	201 (42.68)	149 (31.63)	52 (11.04)	
College or above	159 (2.90)	44 (27.67)	25 (15.72)	49 (30.82)	41 (25.79)	
Residence, *n* (%)						**0.030**
Rural	932 (17.01)	194 (20.82)	177 (18.99)	281 (30.15)	280 (30.04)	
Urban	4,547 (82.99)	1,108 (24.37)	933 (20.52)	1,290 (28.37)	1,216 (26.74)	
Living arrangement, *n* (%)						**<0.001**
Living with family	5,365 (97.92)	1,260 (23.49)	1,078 (20.09)	1,551 (28.91)	1,476 (27.51)	
Others	114 (2.08)	42 (36.84)	32 (28.07)	20 (17.54)	20 (17.54)	
Economic situation, *n* (%)						**<0.001**
Low	1,936 (35.33)	381 (19.68)	554 (28.62)	615 (31.77)	386 (19.94)	
Common	2,091 (38.16)	514 (24.58)	577 (27.59)	581 (27.79)	419 (20.04)	
High	1,452 (26.50)	407 (28.03)	365 (25.14)	375 (25.83)	305 (21.01)	
Smoking, *n* (%)						0.559
No	5,263 (96.06)	1,252 (23.79)	1,069 (20.31)	1,514 (28.77)	1,428 (27.13)	
Yes	216 (3.94)	50 (23.15)	41 (18.98)	57 (26.39)	68 (31.48)	
Drinking, *n* (%)						0.312
No	3,608 (65.85)	833 (23.09)	742 (20.57)	1,053 (29.19)	980 (27.16)	
Yes	1,871 (34.15)	469 (25.07)	368 (19.67)	518 (27.69)	516 (27.58)	
Exercise, *n* (%)						**<0.001**
No	418 (7.63)	77 (18.42)	70 (16.75)	115 (27.51)	156 (37.32)	
Low intensity	1,511 (27.58)	276 (18.27)	281 (18.60)	449 (29.72)	505 (33.42)	
Average intensity	1,655 (30.21)	402 (24.29)	353 (21.33)	474 (28.64)	426 (25.74)	
High intensity	1,895 (34.59)	547 (28.87)	406 (21.42)	533 (28.13)	409 (21.58)	
Self-rated health, *n* (%)						**0.015**
Good	3,618 (66.03)	886 (24.49)	762 (21.06)	1,016 (28.08)	954 (26.37)	
Bad	1,861 (33.97)	416 (22.35)	348 (18.70)	555 (29.82)	542 (29.12)	
Life satisfaction, *n* (%)						**<0.001**
Satisfaction	4,730 (86.33)	1,077 (22.77)	966 (20.42)	1,353 (28.60)	1,334 (28.20)	
Dissatisfaction	749 (13.67)	225 (30.04)	144 (19.23)	218 (29.11)	162 (21.63)	
Cognitive impairment, *n* (%)						**<0.001**
No	3,657 (66.75)	1,193 (32.62)	976 (26.69)	784 (21.44)	704 (19.25)	
Yes	1,822 (33.25)	109 (5.98)	134 (7.35)	787 (43.19)	792 (43.47)	
Depressive symptom, *n* (%)						0.290
No	3,257 (59.45)	751 (23.06)	655 (20.11)	935 (28.71)	916 (28.12)	
Yes	2,222 (40.55)	551 (24.80)	455 (20.48)	636 (28.62)	580 (26.10)	
Hypertension, *n* (%)						0.554
No	4,834 (88.23)	1,163 (24.06)	975 (20.17)	1,378 (28.51)	1,318 (27.27)	
Yes	645 (11.77)	139 (21.55)	135 (20.93)	193 (29.92)	178 (27.60)	
Diabetes, *n* (%)						0.059
No	5,133 (93.68)	1,239 (24.14)	1,040 (20.26)	1,467 (28.58)	1,387 (27.02)	
Yes	346 (6.32)	63 (18.21)	70 (20.23)	104 (30.06)	109 (31.50)	
Stroke, *n* (%)						**<0.001**
No	5,166 (94.29)	1,263 (24.45)	1,060 (20.52)	1,469 (28.44)	1,374 (26.60)	
Yes	313 (5.71)	39 (12.46)	50 (15.97)	102 (32.59)	122 (38.98)	
Osteoarthritis, *n* (%)						0.125
No	5,005 (91.35)	1,185 (23.68)	1,001 (20.00)	1,456 (29.09)	1,363 (27.23)	
Yes	474 (8.65)	117 (24.68)	109 (23.00)	115 (24.26)	133 (28.06)	
Altitude, *n* (%)						**<0.001**
<500 m	1,583 (28.89)	330 (20.85)	457 (28.87)	463 (29.25)	333 (21.04)	
1,000 m-2,000 m	3,217 (58.72)	760 (23.62)	854 (26.55)	938 (29.16)	665 (20.67)	
>4,000 m	679 (12.39)	212 (31.22)	185 (27.25)	170 (25.04)	112 (16.49)	
Famine severity, *n* (%)						0.525
Less severe	2,091 (38.16)	487 (23.29)	439 (20.99)	610 (29.17)	555 (26.54)	
Serious	3,388 (61.84)	815 (24.06)	671 (19.81)	961 (28.36)	941 (27.77)	

F: ANOVA, χ^2^, Chi-square test; SD, Standard deviation. Bold values indicate statistical significance (*p* < 0.05).

A total of 4,513 participants (82.37%) had no disability, with 7.83 and 9.80% of participants being classified as having mild and severe disability, respectively (Supplementary Table 1). ANOVA results revealed a significant association between age and disability status (*p* < 0.05). The mean ages of participants with no disability, mild disability, and severe disability were 59.98 ± 5.40, 60.92 ± 5.13, and 61.53 ± 5.10 years old, respectively. Chi-square tests indicated that gender, education level, residence, economic situation, drinking, exercise, self-rated health, life satisfaction, cognitive impairment, depressive symptom, diabetes, stroke, altitude and famine severity were significantly associated with disability status (*p* < 0.05). The prevalence of both mild and severe disability was higher among female participants compared to males. Urban participants had higher rates of mild and severe disability than those in rural areas. Additionally, individuals from less severely affected famine regions had a higher prevalence of both mild and severe disability compared to those from more severely affected regions.

### 3.2 Association between EFE and physical function in adults

Table 2 presents the association between EFE and physical disability. All logistic regression models we constructed passed the Hosmer-Lemeshow test, and no linear relationship was found between continuous independent variables and the log-odds of the outcome. After adjusting for all covariates, we found that older adults exposed to famine during the school-age period had a 1.51-fold increased risk of restrictions in BADL compared to the unexposed group (OR = 1.51, 95% CI: 1.03–2.21, *p* < 0.05). Additionally, older individuals exposed to famine during the fetal period (OR = 1.30, 95% CI: 1.01–1.69, *p* < 0.05) and the school-age period (OR = 1.32, 95% CI: 1.03–1.70, *p* < 0.05) had 1.30 and 1.32 times higher risk, respectively, of restrictions in IADL compared to those unexposed. Compared with individuals unexposed to famine, those exposed during the fetal period had a 57% higher risk of severe disability (OR = 1.57, 95% CI: 1.14–2.16, *p* < 0.05). Results from the ordinal logistic regression model also indicated that early-life famine exposure was significantly associated with increased disability in later life (*β* = 0.34, 95% CI: 0.10–0.58, *p* < 0.05).

**Table 2:** Association of EFE and physical function.

Model	OR (95% CI)			
	Non- exposed	Fetal exposed	Preschool exposed	School-age exposed
BADL restriction^a^				
Model 1	Ref.	1.30 (0.89,1.89)	1.49 (1.06,2.10)*	2.18 (1.57,3.02)***
Model 2	Ref.	1.31 (0.90,2.20)	1.11 (0.75,1.63)	1.51 (1.03,2.21)*
IADL restriction^a^				
Model 1	Ref.	1.22 (0.96,1.55)	1.48 (1.19,1.83)***	1.80 (1.46,2.23)***
Model 2	Ref.	1.30 (1.01,1.69)*	1.15 (0.89,1.47)	1.32 (1.03,1.70)*
Disability^b^				
Mild disability vs. no disability				
Model 1	Ref.	1.71 (1.28,2.28)***	1.29 (0.94,1.77)	1.43 (1.06,1.91)*
Model 2	Ref.	1.24 (0.89,1.73)	1.36 (0.96,1.90)	1.05 (0.76,1.46)
Severe disability vs. no disability				
Model 1	Ref.	2.14 (1.65,2.77)***	1.21 (0.89,1.64)	1.51 (1.15,1.98)**
Model 2	Ref.	1.57 (1.14,2.16)**	1.32 (0.94,1.84)	1.17 (0.85,1.60)
	β (95% CI)			
Disability^c^	Non- exposed	Fetal exposed	Preschool exposed	School-age exposed
Model 1	Ref.	0.69 (0.47,0.87)***	0.22 (−0.01,0.44)	0.38 (0.18,0.59)***
Model 2	Ref.	0.34 (0.10,0.58)**	0.28 (−0.03,0.53)	0.10 (−0.14,0.34)

BADL: Basic Activities of Daily Living; IADL: Instrumental Activities of Daily Living; Ref.: Reference; *p < 0.05; **p < 0.01; ***p < 0.001. ^a^Binary Logistic Regression. ^b^Multinomial Logistic Regression. ^c^Ordinal Logistic Regression. Model 1: Crude model. Model 2: Adjusted for age, gender, living arrangement, residence, education level, economic situation, smoking, drinking, exercise, self-rated health, life satisfaction, altitude, famine severity, cognitive impairment, depressive symptoms, hypertension, diabetes, stroke, osteoarthritis.

### 3.3 Association between EFE and physical function in Chinese people, grouped by gender, residence and famine severity

Table 3 shows the association between EFE and physical disability after stratifying by gender. After adjusting for all covariates, older men exposed to famine during the fetal period had a 1.69-fold increased risk of severe disability compared to those unexposed (OR = 1.69, 95% CI: 1.03–2.60). Among women, exposure during the school-age period was associated with a 1.52-fold increased risk of severe disability compared to those unexposed (OR = 1.52, 95% CI: 1.05–2.19, *p* < 0.05). School-age famine exposure was significantly associated with restrictions in BADL among men (OR = 1.71, 95% CI: 1.01–2.89, *p* < 0.05) and restrictions in IADL among women (OR = 1.42, 95% CI: 1.01–1.99, *p* < 0.05).

**Table 3:** Association of EFE and physical function, grouped by gender.

Model	OR (95% CI)			
	Non- exposed	Fetal exposed	Preschool exposed	School-age exposed
Males				
BADL restriction				
Model 1	Ref.	1.01 (0.57,1.81)	1.41 (0.86,2.31)	2.14 (1.35,3.38)**
Model 2	Ref.	1.17 (0.63,2.16)	1.29 (0.74,2.22)	1.71 (1.01,2.89)*
IADL restriction				
Model 1	Ref.	1.20 (0.82,1.76)	1.63 (1.17,2.27)**	1.90 (1.38,2.61)***
Model 2	Ref.	1.37 (0.89,2.10)	1.22 (0.83,1.81)	1.13 (0.77,1.66)
Mild disability vs. no disability				
Model 1	Ref.	1.48 (0.92,2.36)	1.35 (0.79,2.29)	1.55 (0.97,2.49)
Model 2	Ref.	0.87 (0.51,1.47)	1.45 (0.83,2.53)	1.13 (0.68,1.91)
Severe disability vs. no disability				
Model 1	Ref.	2.31 (1.58,3.39)***	1.06 (0.65,1.72)	1.60 (1.07,2.41)*
Model 2	Ref.	1.69 (1.03,2.60)*	1.26 (0.73,2.16)	1.35 (0.83,2.18)
Females				
BADL restriction				
Model 1	Ref.	1.56 (0.94,2.58)	1.56 (0.97,2.50)	2.17 (1.35,3.48)**
Model 2	Ref.	1.70 (1.00,2.90)*	1.030 (0.59,1.79)	1.32 (0.75,2.32)
IADL restriction				
Model 1	Ref.	1.23 (0.91,1.66)	1.39 (1.05,1.84)*	1.87 (1.40,2.48)***
Model 2	Ref.	1.31 (0.95,1.82)	1.10 (0.788,1.52)	1.42 (1.01,1.99)*
Mild disability vs. no disability				
Model 1	Ref.	0.95 (0.58,1.55)	0.90 (0.56,1.46)	0.91 (0.59,1.40)
Model 2	Ref.	0.91 (0.52,1.62)	0.86 (0.49,1.49)	0.95 (0.60,1.49)
Severe disability vs. no disability				
Model 1	Ref.	2.04 (1.42,2.94)***	1.55 (1.08,2.22)*	1.41 (1.02,1.96)*
Model 2	Ref.	1.51 (0.96,2.37)	1.06 (0.68,1.65)	1.52 (1.05,2.19)*

BADL, Basic activities of daily living; IADL, Instrumental activities of daily living; Ref., Reference. *: *p* < 0.05; **: *p* < 0.01; ***: *p* < 0.001. Model 1: Crude model. Model 2: Adjusted for age, gender, living arrangement, residence, education level, economic situation, smoking, drinking, exercise, self- rated health, life satisfaction, altitude, famine severity, cognitive impairment, depressive symptom, hypertension, diabetes, stroke, osteoarthritis.

Table 4 presents the association between EFE and physical disability stratified by residence. After controlling for all covariates, older adults in rural areas who were exposed to famine during the fetal period had a 1.65-fold increased risk of severe disability compared to those unexposed (OR = 1.65, 95% CI: 1.34–2.77, *p* < 0.05). In urban areas, individuals exposed during the preschool period had a 1.63-fold increased risk of mild disability (OR = 1.63, 95% CI: 1.23–2.25, *p* < 0.05).

**Table 4:** Association of EFE and physical function, grouped by residence.

Model	OR (95% CI)			
	Non- exposed	Fetal exposed	Preschool exposed	School-age exposed
Rural				
BADL restriction				
Model 1	Ref.	1.16 (0.69,1.72)	1.04 (0.71,1.56)	1.88 (1.18,2.18)**
Model 2	Ref.	1.35 (0.49,2.57)	0.73 (0.67,1.68)	1.14 (0.72,1.36)
IADL restriction				
Model 1	Ref.	1.15 (0.74,1.87)	1.77 (1.15,2.26)**	1.58 (1.18,2.46)**
Model 2	Ref.	1.21 (0.89,1.36)	1.26 (0.83,1.40)	1.23 (0.72,1.89)
Mild disability vs. no disability				
Model 1	Ref.	1.63 (0.39,1.94)	1.36 (0.75,2.28)	1.30 (1.07,2.23)*
Model 2	Ref.	1.23 (0.57,3.64)	1.23 (0.67,2.34)	1.48 (0.94,2.02)
Severe disability vs. no disability				
Model 1	Ref.	2.37 (1.12,3.33)***	1.91 (1.13,2.55)**	1.39 (1.10,2.52)*
Model 2	Ref.	1.65 (1.34,2.77)*	1.24 (0.85,1.87)	1.36 (0.77,2.19)
Urban				
BADL restriction				
Model 1	Ref.	1.46 (0.84,2.36)	1.44 (0.81,2.10)	2.33 (1.66,3.43)***
Model 2	Ref.	1.42 (0.97,2.90)	1.55 (0.86,2.42)	1.56 (1.08,2.99)*
IADL restriction				
Model 1	Ref.	1.19 (0.95,1.48)	1.38 (1.00,1.58)*	1.39 (1.17,2.50)***
Model 2	Ref.	1.39 (0.88,1.77)	1.02 (0.89,1.49)	1.49 (0.67,1.94)
Mild disability vs. no disability				
Model 1	Ref.	1.74 (1.14,2.00)***	1.68 (0.97,2.23)	1.49 (0.87,1.92)
Model 2	Ref.	1.35 (0.74,2.35)	1.63 (1.23,2.25)*	1.27 (0.34,1.63)
Severe disability vs. no disability				
Model 1	Ref.	2.21 (1.59,2.48)***	1.67 (0.37,1.74)	1.57 (1.27,2.74)*
Model 2	Ref.	1.63 (0.69,2.37)	1.26 (0.59,2.86)	0.90 (0.68,1.57)

BADL, Basic activities of daily living; IADL, Instrumental activities of daily living; Ref., Reference. *: *p* < 0.05; **: *p* < 0.01; ***: *p* < 0.001. Model 1: Crude model. Model 2: Adjusted for age, gender, living arrangement, residence, education level, economic situation, smoking, drinking, exercise, self- rated health, life satisfaction, altitude, famine severity, cognitive impairment, depressive symptom, hypertension, diabetes, stroke, osteoarthritis.

Table 5 shows the association between EFE and physical disability after stratification by famine severity across mainland China. After adjusting for all covariates, in regions with less severe famine exposure, those exposed to famine during the fetal period had a 1.66 times higher probability of experiencing severe disability compared to those unexposed (OR = 1.66, 95% CI: 1.01–2.71, *p* < 0.05). In regions with more severe famine exposure, school-age famine exposure was significantly associated with limitations in BADL (OR = 1.75, 95% CI: 1.03–2.97, *p* < 0.05), while those exposed to famine during the preschool period had a 1.57 times higher probability of experiencing mild disability compared to those unexposed (OR = 1.57, 95% CI: 1.02–2.41, *p* < 0.05).

**Table 5:** Association of EFE and physical function, grouped by famine severity.

Model	OR (95% CI)			
	Non- exposed	Fetal exposed	Preschool exposed	School-age exposed
Less severely affected famine area				
BADL restriction				
Model 1	Ref.	1.16 (0.67,2.02)	1.05 (0.62,1.77)	1.91 (1.18,3.10)**
Model 2	Ref.	1.25 (0.69,2.28)	0.78 (0.43,1.43)	1.33 (0.75,2.36)
IADL restriction				
Model 1	Ref.	1.15 (0.79,1.67)	1.55 (1.11,2.16)**	1.63 (1.17,2.29)**
Model 2	Ref.	1.27 (0.84,1.91)	1.27 (0.86,1.90)	1.18 (0.78,1.78)
Mild disability vs. no disability				
Model 1	Ref.	1.63 (0.91,2.94)	1.38 (0.77,2.49)	1.80 (1.09,2.97)*
Model 2	Ref.	1.75 (0.90,3.39)	1.44 (0.74,2.72)	1.68 (0.99,2.84)
Severe disability vs. no disability				
Model 1	Ref.	2.16 (1.44,3.24)***	1.77 (1.19,2.64)**	1.55 (1.09,2.21)*
Model 2	Ref.	1.66 (1.01,2.71)*	1.22 (0.75,1.98)	1.41 (0.94,2.10)
Severely affected famine area				
BADL restriction				
Model 1	Ref.	1.41 (0.84,2.36)	1.91 (1.21,3.01)**	2.13 (1.56,3.80)***
Model 2	Ref.	1.49 (0.85,2.60)	1.46 (0.86,2.46)	1.75 (1.03,2.97)*
IADL restriction				
Model 1	Ref.	1.26 (0.93,1.72)	1.42 (1.07,1.88)*	1.92 (1.47,2.52)***
Model 2	Ref.	1.33 (0.95,1.87)	1.02 (0.73,1.42)	1.31 (0.94,1.82)
Mild disability vs. no disability				
Model 1	Ref.	2.00 (1.38,2.89)***	1.49 (0.98,2.25)	1.33 (0.90,1.97)
Model 2	Ref.	1.50 (0.98,2.29)	1.57 (1.02,2.41)*	1.01 (0.65,1.57)
Severe disability vs. no disability				
Model 1	Ref.	2.12 (1.51,2.98)***	1.19 (0.80,1.78)	1.59 (1.12,2.26)*
Model 2	Ref.	1.52 (0.99,2.33)	1.29 (0.83,2.03)	0.18 (0.77,1.80)

BADL, Basic activities of daily living; IADL, Instrumental activities of daily living; Ref.: Reference. *: *p* < 0.05; **: *p* < 0.01; ***: *p* < 0.001. Model 1: Crude model. Model 2: Adjusted for age, gender, living arrangement, residence, education level, economic situation, smoking, drinking, exercise, self- rated health, life satisfaction, altitude, famine severity, cognitive impairment, depressive symptom, hypertension, diabetes, stroke, osteoarthritis.

### 3.4 Sensitivity analysis

This study conducted two sensitivity analyses. First, we performed multiple imputation using chained equations (MICE) for missing variables in the participants (Supplementary Table 2). Second, we excluded participants with cognitive impairment, and the results consistently indicated a statistically significant association between EFE and physical disability (Supplementary Table 3).

## 4 Discussion

The results of this study showed that fetal famine exposure was significantly associated with severe disability after adjusting for covariates. When stratified by sex, a significant correlation between fetal famine exposure and severe disability was held for males; and a strongly association between school-age famine exposure and severe disability was observed for females. Fetal famine exposure was significantly associated with severe disability among rural participants, whereas preschool famine exposure was significantly associated with mild disability among urban participants. Fetal famine exposure was significantly associated with severe disability among participants from mild famine areas, and preschool-age famine exposure was significantly associated with mild disability among participants from severe famine areas.

Previous studies have confirmed that good child health increases the likelihood of better physical functioning in Chinese adults by 14% (45), which suggests that early life may be a critical period for the development of physical function. However, few studies have directly investigated the association between EFE and physical disability. Our results showed that exposure to famine during fetal life is significantly associated with severe disability in later life. Epigenetics may help explain the underlying mechanisms between the two. Epigenetics reveals genetic imprinting, programming, and reprogramming in early life and increased disease susceptibility in later life (46). The fetus anticipates the environment it will encounter in the future in the mother’s womb and appropriately alters metabolic, physiological and developmental trajectories to maximize postnatal survival (46). However, if the postnatal environment is different than predicted, these adaptations may lead to metabolic and endocrine changes that lead to lifelong changes in body function and structure, and may lead to the onset of disease in later life (47). EFE resulted in the fetus displaying a specific epigenetic state of famine exposure, whereas the Great Chinese Famine lasted only 3 years, after which the rapid development of the new China and the gradual affluence of the people resulted in a different epigenetic state from that of the later growth environment and thus became a predisposing factor for the disease. In addition, in the fetal period, malnutrition alters the expression of certain genes in the hypothalamus that regulate nutrient sensing and energy homeostasis, leading to abnormal growth and metabolic function in adulthood, which partly explains the association between exposure to famine in the fetal period and disability in later life (48). Abnormalities in the development of the musculoskeletal system as a result of EFE may also be closely associated with the onset of disability. The development of the musculoskeletal system may explain this association to some degree. The development of immature muscle fiber depends on protein synthesis and the proliferation of satellite cells, which become quiescent as muscle fiber becomes mature and muscle protein anabolism decreases (46). Early malnutrition directly affects the growth and metabolism of immature muscle fibers. In addition, impaired nutrient delivery to the fetus leads to shifts in blood flow and nutrients, in which situation the organism protects the brain and organs at the expense of muscle growth (49). Early development is a critical window for muscle growth, during which period poor nutrition early in life can lead to inadequate muscle mass, a disease unlikely to fully recover, which can further result in reduced physical function and even disability later in life. A recent study has also demonstrated that EFE is related to potential sarcopenia in adults (11), which is a major contributor to their functional dependency and physical disability (50). In this study, only EFE was observed to be significantly associated with severe disability rather than mild disability. Some studies have seen EFE as a type of adverse childhood experience (51), and the adverse health effects of these adverse experiences accumulate as people age (52). At the same time, aging, accompanied by declining physical functioning, is more likely to manifest as severe disability in old age.

When stratified by sex, we discovered that we found that the significant correlation between fetal famine exposure and severe disability continued to hold in males, which is consistent with the majority of studies supporting the greater vulnerability of males to early adverse environmental effects (53, 54). The placenta provides oxygen and nutrients and facilitates the exchange of substances between the maternal and fetal circulations (55). When maternal malnutrition occurs, placental weight, morphology, vascular development and amino acid transport functions are altered, resulting in impaired nutritional supply to the fetus (56). A study published in *The Lancet* highlights that the first 1,000 days–from conception to a child’s second birthday—constitute a critical window for growth and development (57). Evidence suggests that stunting after this period is largely irreversible, perpetuating an intergenerational cycle of impaired growth and development (58). Fetal exposure to famine may result in undernutrition during this crucial period, thereby adversely affecting lifelong health and productivity (59). Erikson et al. found that boys were larger and grew faster in utero during early pregnancy (60). However, boys have lower placental reserve capacity (the ability to transfer oxygen and nutrients to the fetus), and where nutrients cannot be sourced directly from the mother and need to be sustained through the transfer capacity of the placenta, boys have fewer reserves available than girls of the same weight, meaning that boys are at increased risk of prenatal malnutrition. The same study also suggests that boys are more sensitive than girls to their mothers’ gestational diets during uterine development (61), and that boys may be more likely than girls to exhibit malnutrition when their mothers are exposed to famine. In addition, we observed that school-age famines exposure in females was associated with severe disability. This may be attributed to the lower social status of females in traditional Chinese culture (62, 63). China’s preference for sons over daughters leads to the birth of girls who grow up with less access to food and resources for growth, and thus girls may be thinner and show less resistance to adversity than boys. Girls may be more susceptible to the hazards of school-age famine exposure and thus exhibit severe disabilities later in life. However, the potential mechanisms by which exposure to famine during the fetal period affects disability in middle-aged and older adults and gender differences require further research.

Stratified analyses by residence revealed significant urban–rural disparities in the association between early-life famine exposure and disability risk. Among rural participants, fetal exposure to famine was significantly associated with an increased risk of severe disability. This may be attributable to nutritional deprivation during critical periods of organogenesis, potentially resulting in irreversible structural damage (64). The limited availability of perinatal healthcare resources in rural areas may further exacerbate the severity of these developmental impairments. In contrast, among urban participants, exposure to famine during the preschool period was significantly associated with mild disability. This developmental stage is characterized by rapid physical and neurological growth particularly in the central nervous system (65), marked by both high plasticity and vulnerability; nutritional deficiency during this window may lead to functional impairments. Moreover, more comprehensive systems for child health screening and management in urban areas may facilitate earlier detection and intervention for mild deficits. These findings suggest that the health consequences of famine are modified by sociogeographic context, highlighting the need for taking into consideration regional disparities in launching disability prevention strategies. We also observed that fetal famine exposure was significantly associated with severe disability in mild famine areas, and preschool famine exposure was significantly associated with mild disability in severe famine areas. The harmful effects of severe undernutrition on the organism are severe and long-lasting and may lead to oxidative stress, metabolic disorders, liver disease, kidney disease, and brain damage, which can lead to disability, whereas the effects of mild malnutrition on the organism may not be sufficient to manifest as disability (66). However, the fetal period is an important period of human development and its sensitivity to adverse external stimuli is much higher than that of the preschool period. So exposure to famine, though milder in the fetal period, is significantly associated with the development of severe disability. In contrast, sensitivity to external stimuli is relatively low during the preschool period when the most obvious consequences of nutritional deficiencies are loss of body weight and adipose tissue, as well as micronutrient deficiencies that result in slowed linear growth (67). Based on these findings, we hypothesize that exposure to severe famine during the preschool period may not induce the epigenetic alterations typically associated with fetal-stage exposure, nor substantially impair the development of immature muscle fibers, and thus may manifest primarily as mild disability. Nevertheless, caregiving during the preschool years—particularly adequate early-life nutrition—remains critical for optimal child growth and development. The second critical window of child development (ages 2–5 years old, referred to as the second 1,000 days of life) plays an irreplaceable role in consolidating the developmental gains achieved during the first 1,000 days (68). This period not only strengthens the protective effects of early interventions and mitigates the adverse impacts of environmental risk factors but also offers a developmental compensation mechanism, enabling children who lacked sufficient developmental opportunities in the first 1,000 days to reconstruct a positive trajectory for healthy growth (68).

Little literature has focused on the effects of fetal famine exposure on physical disability in later life, but this cannot be ignored in China where aging is currently a serious problem. With the rapid development, individuals exposed to the Great Chinese Famine during the fetal period are gradually reaching an advanced age, and the prevalence of disability is significantly increasing, which not only affects their quality of life and well-being in their later years, but also limits family labor, leads to a decrease in family income and affects family harmony. In addition, the increasing number of adults with disabilities has also raised the demand for medical services and long-term care. But at the current stage, the number of long-term care resources in China is insufficient, the distribution is unreasonable, and most families can hardly afford long-term medical care. This suggests a need for improvement in the current healthcare system, namely, to reasonably reduce the cost of medical care and rationalize the distribution of medical care resources. Social security organizations should also provide certain social security for families with disabilities to promote family harmony. Additionally, we can try to collect more detailed information about the famine-exposed population to build a prediction model of disability in this population in future research, and take more targeted measures to intervene, to reduce the incidence of disability and improve the quality of life of the middle-aged and older adults.

## 5 Conclusion

This study, through the analysis of large-scale data from CHARLS, revealed a significant association between exposure to famine during early life stages and the risk of disability in middle and older age. Further subgroup analyses by gender, residence and famine severity uncovered the specificity of this association. The findings provide critical evidence for understanding the impact of early-life malnutrition on long-term health outcomes, underscoring the importance for public health policymakers to prioritize nutritional status during early life stages due to its enduring effects on health. Additionally, this study calls upon individuals who have experienced famine to pay attention to their physical health and adopt targeted measures in a timely manner to prevent or delay the onset and progression of disability.

## 6 Limitations

This study has several limitations. First, as an observational study, it cannot establish causal relationships. Second, due to missing data, some participants were excluded from the analysis, which may limit the generalizability of the findings. However, after multiple imputations for missing variables, the significant association between EFE and disability remained, indicating the robustness of the results. Third, as famine exposure occurred in early life and was assessed retrospectively, the findings may be subject to recall bias. Nevertheless, previous studies have demonstrated the reliability of these retrospective measures. Furthermore, after excluding participants with cognitive impairment, the significant association between EFE and disability persisted, further supporting the validity of our findings. Fourth, although we adjusted for a wide range of covariates, including demographic characteristics, lifestyle factors, and health status, some relevant variables (e.g., prior fractures) could not be assessed due to limitations in the questionnaire. Despite these limitations, this is the first study to use nationally representative data to evaluate the impact of the Chinese Great Famine on disability. These important findings enhance our understanding of the long-term consequences of famine on disability in older Chinese adults and provide evidence for sex-specific patterns of disability, which may inform strategies to prevent disability in high-risk populations.

## Data Availability

The datasets presented in this study can be found in online repositories. The names of the repository/repositories and accession number(s) can be found at: http://charls.ccer.edu.cn/charls/.
