# Supplementary material for: Early-life famine exposure increases the risk of subsequent physical disability: evidence from a national population-based survey
Source: Front Public Health. 2025 Jul 9;13:1587680. doi: 10.3389/fpubh.2025.1587680 (PMC12283694; doi:10.3389/fpubh.2025.1587680)
Supplement: Supplementary file 1 [file Data_Sheet_1.docx]

Supplementary Material

# Supplementary Tables

**Supplementary Table 1. Characteristics of Participants by Degree of Disability**

| **Variables** | **Total**  **(n =** 5,479**)** | **No disability**  **(n =** 4,513**)** | **Mild disability**  **(n =** 429**)** | **Severe disability**  **(n =** 537**)** | **P** | |
| --- | --- | --- | --- | --- | --- | --- |
|  |  |  |  |  |  |  |
| Age , Mean ± SD | 60.20 ± 5.38 | 59.98 ± 5.40 | 60.92 ± 5.13 | 61.53 ± 5.10 | **<.001** | |
| Gender , n(%) |  |  |  |  | **<.001** | |
| Male | 2,710 (49.46) | 2,283 (84.24) | 166 (6.13) | 261 (9.63) |  | |
| Female | 2,769 (50.54) | 2,230 (80.53) | 263 (9.50) | 276 (9.97) |  | |
| Education level , n(%) |  |  |  |  | **<.001** | |
| Illiteracy | 3,546 (64.72) | 2,792 (78.74) | 349 (9.84) | 405 (11.42) |  | |
| Primary school or below | 1,303 (23.78) | 1,152 (88.41) | 54 (4.14) | 97 (7.44) |  | |
| Secondary school or below | 471 (8.60) | 425 (90.23) | 20 (4.25) | 26 (5.52) |  | |
| College or above | 159 (2.90) | 144 (90.57) | 6 (3.77) | 9 (5.66) |  | |
| Residence, n(%) |  |  |  |  | **<.001** | |
| Rural | 932 (17.01) | 824 (88.41) | 38 (4.08) | 70 (7.51) |  | |
| Urban | 4,547 (82.99) | 3,689 (81.13) | 391 (8.60) | 467 (10.27) |  | |
| Living arrangement, n(%) |  |  |  |  | 0.580 | |
| Living with family | 5,365 (97.92) | 4,417 (82.33) | 423 (7.88) | 525 (9.79) |  | |
| Others | 114 (2.08) | 96 (84.21) | 6 (5.26) | 12 (10.53) |  | |
| Economic situation ,n(%） |  |  |  |  | **<.001** |  |
| Low | 1,936 (35.33) | 1,526 (78.82) | 229 (11.83) | 181 (9.35) |  | |
| Common | 2,091 (38.16) | 1,749 (83.64) | 185 (8.85) | 157 (7.51) |  | |
| High | 1,452 (26.50) | 1,238 (85.26) | 123 (8.47) | 91 (6.27) |  | |
| Smoking, n(%) |  |  |  |  | 0.616 | |
| No | 5,263 (96.06) | 4,337 (82.41) | 414 (7.87) | 512 (9.73) |  | |
| Yes | 216 (3.94) | 176 (81.48) | 15 (6.94) | 25 (11.57) |  | |
| Drinking, n(%) |  |  |  |  | **<.001** | |
| No | 3,608 (65.85) | 2,883 (79.91) | 319 (8.84) | 406 (11.25) |  | |
| Yes | 1,871 (34.15) | 1,630 (87.12) | 110 (5.88) | 131 (7.00) |  | |
| Exercise, n(%) |  |  |  |  | **<.001** | |
| No | 418 (7.63) | 268 (64.11) | 34 (8.13) | 116 (27.75) |  | |
| Low intensity | 1,511 (27.58) | 1,176 (77.83) | 133 (8.80) | 202 (13.37) |  | |
| Average intensity | 1,655 (30.21) | 1,432 (86.53) | 120 (7.25) | 103 (6.22) |  | |
| High intensity | 1,895 (34.59) | 1,637 (86.39) | 142 (7.49) | 116 (6.12) |  | |
| Self-rated health, n(%) |  |  |  |  | **<.001** | |
| Good | 3,618 (66.03) | 3,269 (90.35) | 205 (5.67) | 144 (3.98) |  | |
| Bad | 1,861 (33.97) | 1,244 (66.85) | 224 (12.04) | 393 (21.12) |  | |
| Life satisfaction, n(%) |  |  |  |  | **<.001** | |
| Satisfaction | 4,730 (86.33) | 4,014 (84.86) | 333 (7.04) | 383 (8.10) |  | |
| Dissatisfaction | 749 (13.67) | 499 (66.62) | 96 (12.82) | 154 (20.56) |  | |
| Cognitive impairment, n(%) |  |  |  |  | **<.001** | |
| No | 3,657 (66.75) | 3,133 (85.67) | 228 (6.23) | 296 (8.09) |  | |
| Yes | 1,822 (33.25) | 1,380 (75.74) | 201 (11.03) | 241 (13.23) |  | |
| Depressive symptom , n(%) |  |  |  |  | **<.001** | |
| No | 3,257 (59.45) | 2,919 (89.62) | 193 (5.93) | 145 (4.45) |  | |
| Yes | 2,222 (40.55) | 1,594 (71.74) | 236 (10.62) | 392 (17.64) |  | |
| Hypertension , n(%) |  |  |  |  | 0.060 | |
| No | 4,834 (88.23) | 3,996 (82.66) | 381 (7.88) | 457 (9.45) |  | |
| Yes | 645 (11.77) | 517 (80.16) | 48 (7.44) | 80 (12.40) |  | |
| Diabetes , n(%) |  |  |  |  | **0.021** | |
| No | 5,133 (93.68) | 4,247 (82.74) | 394 (7.68) | 492 (9.59) |  | |
| Yes | 346 (6.32) | 266 (76.88) | 35 (10.12) | 45 (13.01) |  | |
| Stroke , n(%) |  |  |  |  | **<.001** | |
| No | 5,166 (94.29) | 4,319 (83.60) | 386 (7.47) | 461 (8.92) |  | |
| Yes | 313 (5.71) | 194 (61.98) | 43 (13.74) | 76 (24.28) |  | |
| Osteoarthritis ,n(%) |  |  |  |  | 0.747 | |
| No | 5,005 (91.35) | 4,128 (82.48) | 388 (7.75) | 489 (9.77) |  | |
| Yes | 474 (8.65) | 385 (81.22) | 41 (8.65) | 48 (10.13) |  | |
| Altitude , n(%) |  |  |  |  | **0.007** | |
| <500m | 1,583 (28.89) | 1,343 (84.84) | 120 (7.58) | 120 (7.58) |  | |
| 1,000m-2,000m | 3,217 (58.72) | 2,627 (81.66) | 339 (10.54) | 251 (7.80) |  | |
| >4,000m | 679 (12.39) | 543 (79.97) | 78 (11.49) | 58 (8.54) |  | |
| Famine severity, n(%) |  |  |  |  | 0.100 | |
| Less severe | 2,091 (38.16) | 1,694 (81.01) | 172 (8.23) | 225 (10.76) |  | |
| Serious | 3,388 (61.84) | 2,819 (83.21) | 257 (7.59) | 312 (9.21) |  | |

Note :F: ANOVA, χ²: Chi-square test;SD: Standard Deviation

**Supplementary Table 2. Association of EFE and Physical Function -Multiple Linear Imputation of Missing Values**(n=6,629)

| Model | OR(95%CI) | | | |
| --- | --- | --- | --- | --- |
|  | Non- exposed | Fetal exposed | Preschool exposed | School-age exposed |
| BADL restriction |  |  |  |  |
| Model 1 | Ref. | 1.227(0.879,1.712) | 1.645(1.228,2.203)** | **2.436(1.842,3.223)***** |
| Model 2 | Ref. | 1.188(0.832,1.698) | 1.391(0.999,1.938) | **1.787(1.291,2.474)***** |
| IADL restriction |  |  |  |  |
| Model 1 | Ref. | 1.211(0.980,1.495) | 1.524(1.263,1.839)*** | **1.999(1.664,2.402)***** |
| Model 2 | Ref. | 1.233(0.978,1.555) | 1.213(0.972,1.512) | **1.420(1.139,1.771)**** |
| Mild disability vs. no disability |  |  |  |  |
| Model 1 | Ref. | 1.886(1.451,2.452)*** | 1.226(0.941,1.705) | 1.440(1.100,1.884)** |
| Model 2 | Ref. | 1.321(0.978,1.784) | 1.280(0.940,1.743) | 1.045(0.773,1.413) |
| Severe disability vs. no disability |  |  |  |  |
| Model 1 | Ref. | **2.265(1.813,2.829)***** | 1.217(0.938,1.578) | **1.585(1.259,1.994)***** |
| Model 2 | Ref. | **1.712(1.309,2.239)***** | 1.270(0.952,1.695) | **1.370(1.045,1.796)*** |

Note:BADL:Basic Activities of Daily Living;IADL:Instrumental Activities of Daily Living;Ref.:Reference

** :P < 0.01,***:P< 0.001

Model 1:Crude model

Model 2:Adjusted for age,gender, living arrangement,residence, education level, economic situation, smoking, drinking, exercise ,self- rated health, life satisfaction,altitude,famine severity, cognitive impairment, depressive symptom, hypertension, diabetes, stroke, osteoarthritis.

**Supplementary Table 3. Association of EFE and Physical Function -Participants with Cognitive Impairment were Excluded**（n=3,657）

| Model | OR(95%CI) | | | |
| --- | --- | --- | --- | --- |
|  | Non- exposed | Fetal exposed | Preschool exposed | School-age exposed |
| BADL restriction |  |  |  |  |
| Model 1 | Ref. | 1.261(0.834,1.895) | 1.048(0.667,1.648) | **1.706(1.129,2.580)*** |
| Model 2 | Ref. | 1.292(0.843,1.982) | 1.033(0.640,1.667) | **1.744(1.115,2.727)*** |
| IADL restriction |  |  |  |  |
| Model 1 | Ref. | 1.155(0.894,1.492) | 1.083(0.822,1.427) | **1.344(1.024,1.764)*** |
| Model 2 | Ref. | 1.188(0.901,1.568) | 1.160(0.857,1.568) | **1.492(1.102,2.019)*** |
| Mild disability vs. no disability |  |  |  |  |
| Model 1 | Ref. | 1.144(0.773,1.692) | 1.271(0.900,1.794) | 1.002(0.680,1.477) |
| Model 2 | Ref. | 1.215(0.798,1.850) | 1.425(0.991,2.050) | 1.113(0.740,1.673) |
| Severe disability vs. no disability |  |  |  |  |
| Model 1 | Ref. | **1.728(1.248,2.392)**** | 1.130(0.815,1.568) | **1.143(0.810,1.615)** |
| Model 2 | Ref. | **1.545(1.058,2.255)*** | 1.269(0.881,1.829) | **1.164(0.789,1.717)** |

Note:BADL:Basic Activities of Daily Living;IADL:Instrumental Activities of Daily Living;Ref.:Reference

* :P < 0.05,** :P < 0.01

Model 1:Crude model

Model 2:Adjusted for age,gender, living arrangement,residence, education level, economic situation, smoking, drinking, exercise ,self- rated health, life satisfaction,altitude,famine severity, cognitive impairment, depressive symptom, hypertension, diabetes, stroke, osteoarthritis.
